# Local extinction of a parasite of Magellanic penguins? The effect of a warming hotspot on a ‘cold’ trematode

**DOI:** 10.1017/S0031182025000216

**Published:** 2025-03

**Authors:** Paula Marcotegui, Matias Merlo, Manuel Marcial Irigoitia, María Paz Gutiérrez, Claudio Buratti, Juan Pablo Seco Pon, Manuela Parietti, Juan Tomás Timi

**Affiliations:** 1 Laboratorio de Ictioparasitología, Instituto de Investigaciones Marinas y Costeras (IIMyC), Facultad de Ciencias Exactas y Naturales, Universidad Nacional de Mar del Plata-CONICET, Mar del Plata, Argentina; 2 Instituto Nacional de Investigación y Desarrollo Pesquero (INIDEP), Mar del Plata, Argentina; 3 Laboratorio de Vertebrados, Instituto de Investigaciones Marinas y Costeras (IIMyC), Facultad de Ciencias Exactas y Naturales, Universidad Nacional de Mar del Plata-CONICET, Mar del Plata, Argentina

**Keywords:** ecological indicators, global change, ocean warming, parasite extinction

## Abstract

It is often postulated that natural systems are expected to suffer an increasing risk of infectious disease outbreaks as climate change accelerates. In the northern Argentine Sea, the rise of ocean temperature has produced a tropicalization of demersal megafauna since 2013. This rapidly warming hotspot provides an excellent model to test whether fish parasites have increased, declined, or remained stable in the region. *Cardiocephaloides physalis* a parasite of penguins *Spheniscus magellanicus* as adult and suspected to parasitize anchovies *Engraulis anchoita* as larvae is here used to compare their occurrence and abundance between samples composed by 1752 fish of variable age caught at different latitudes during 1993–1995 and 2022 and between 20 juvenile birds and literature data. In the present work, the identity of metacercariae as *C. physalis* is confirmed genetically, as well as a net decline of population parameters of the parasite to its effective disappearance in anchovies from northern areas and to extremely low levels in fish from southern regions and penguins. After analysing possible causes for such changes in a scenario of rapid regional tropicalization, a direct effect of increasing temperature on parasites arose as the main causal candidate for the observed decline in their populations over the last decades. Beyond the biological and ecological consequences of global change on them, parasites offer excellent systems for measuring and monitoring such effects. The almost local extinction of *C. physalis* in a marine hotspot of global warming seems to be one of the first examples of such processes.

## Graphical abstract

**Figure:**
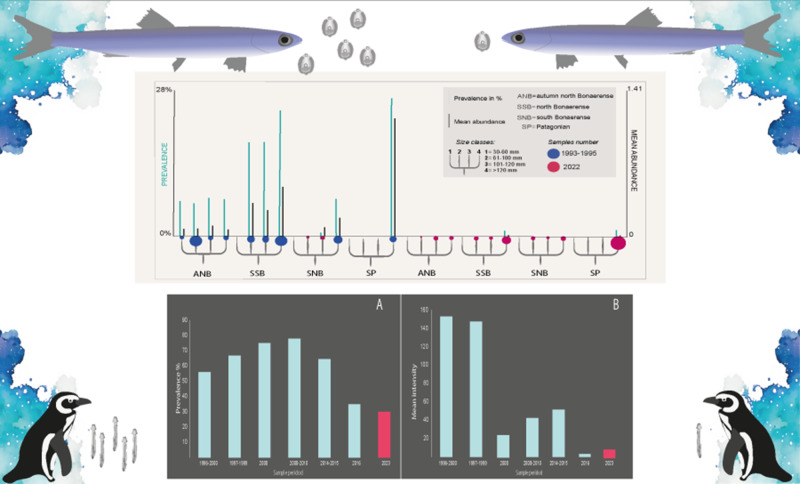

## Introduction

During the last few decades, ongoing global changes have been affecting dramatically the structure and composition of parasite assemblages (Daszak et al., 2000; Marcogliese, 2023). By compromising host resistance and increasing opportunistic diseases, climate change may shift the distribution of either hosts or parasites, consequently affecting populations and communities of free-living organisms (Marcogliese, 2001; Poulin, 2006). In addition to modifying parasite distribution and host susceptibility to extinction, increased temperatures, one of the components of climate change, may increase parasite development and transmission rates and the number of generations per year (Harvell et al., 2002; Poulin, 2006) and disrupt hosts ability to cope with thermal stress (Hector et al., 2019). For these reasons, in general, predictions hypothesise increased probabilities for hosts facing more and/or new parasites, giving rise to the building of new communities (Bordes and Morand, 2009). This is probably due to the majority of studies having focused on virulent pathogens that could become dominant in a warmer world, mainly those of human health concern (Altizer et al., 2013; Cizauskas et al., 2017). Indeed, it has been postulated that even those hosts adapted to cooler or milder climates are expected to suffer an increasing risk of infectious disease outbreaks as climate change accelerates (Cohen et al., 2020). As an example, increased parasitism in cold climes has been recorded for several host-parasite systems in arctic vertebrates, where climate change is taking place more rapidly and severely (Gilg et al., 2012).

However, given the complex link between climate and parasitism, climate change may not always lead to a net increase in the abundance or geographic distribution of parasitism. Upper and lower limits to temperature tolerance may produce shifts, instead of increases or decreases, in species distribution (Lafferty, 2009; Rohr and Cohen, 2020). Also, although warm temperatures can accelerate metabolic rates, allowing increased activity, growth, development and reproduction, faster metabolism requires higher food consumption rates to maintain a positive energy balance, which can decrease survivorship as temperature increases, in particular for non-feeding free-living stages (Lafferty, 2009).

During the last three decades, and at a global scale, a pronounced warming has been recorded for each of the subtropical western boundary currents in the oceans, including the Brazil Current in the Southwestern Atlantic (Johnson and Lyman, 2020). In this region, discrete marine ‘hotspots’ have been identified along the path of the Brazil Current, the Brazil-Malvinas Confluence and the Río de la Plata (Hobday and Pecl, 2014; Franco et al., 2020a). Indeed, on the continental shelf of southern Brazil, Uruguay and northern Argentina, the rise of sea temperatures has impacted the distribution of several fisheries and the *composition* of the captures at regional scale (Franco et al., 2020b), producing a tropicalization of demersal megafauna in this region since 2013 (Gianelli et al., 2019; Alvarez Perez and Sant’Ana, 2022), with many fishery resources displaying different degrees of sensitivity to climate change (Gianelli et al., 2023). This rapidly warming hotspot, encompassing the northern Argentine Sea (Hobday and Pecl, 2014), provides an excellent model to test whether fish parasites have increased, declined, or remained stable, owing to the thermal change undergone during a relatively short period of time.

*Cardiocephaloides* is a cosmopolitan genus of the family Strigeidae (Digenea, Diplostomida), composed by seven species, most of them parasites of larid birds, with only *C. physalis* parasitizing penguins, including its type host *Spheniscus magellanicus* in coastal regions of South America, from Brazil to Peru, as well as from *Spheniscus humboldti* from Chile and Peru and *Spheniscus demersus* from South Africa (Achatz et al., 2020). Other marine birds such as gulls, cormorants, and sooty shearwaters *Ardenna griseus* from Peru have been also reported hosting this parasite species (Achatz et al., 2020). The conspecificity of specimens from South America and their South African Atlantic counterparts, identified as *C. physalis*, has been recently proposed based on 28S rDNA sequence data (Achatz et al., 2020). Also, metacercariae from the eyes of South African sardines *Sardinops sagax* (Clupeidae) and *Clinus superciliosus* (Clinidae), both from South Africa, were confirmed as belonging to this species after sequencing 28S rDNA, ITS2 rDNA-region and COI mtDNA (Ukomadu, 2017; Vermaak et al., 2021).

In the Argentine Sea, metacercariae from the eyes of Argentine anchovies, *Engraulis anchoita*, were recorded as *Cardiocephaloides* sp. (Timi et al., 1999) at relatively high prevalence (6-18%) and intensities between 1 and 23 worms per fish, in anchovies caught between 34º and 46º S (Timi et al., 1999). In adult fishes, the prevalence of this species increased southwards, towards north Patagonian waters, where a discrete anchovy population inhabits (Timi, 2003), indicating a preference for colder waters. Metacercariae recorded by Timi et al. (1999) are supposed to belong to *Cardiocephaloides physalis* due to adults of this species are known for parasitizing *S. magellanicus* in South America (González Acuña et al., 2008; Díaz et al., 2010). Although the conspecificity of larvae and adult parasites requires molecular corroboration to derive proper conclusions, quantitative data of adult parasites in Magellanic penguins have also been available along the Argentine and Brazilian coasts since 1996 (Table 1).

**Table 1.** Values of prevalence (P), mean abundance (MA), and mean intensity (MI) of *Cardiocephaloides physalis* parasitizing *Spheniscus magellanicus*

Year	Locality/s	Examined birds (juvenile/adults)	Prevalence/mean abundance/mean intensity	Reference
1997–1999	Península Valdés, Argentina	4J/5A	66.7/-/147.5	Pazos et al., 2003
1996–2000	Península Valdés, Argentina	27	56/-/153	Díaz et al., 2010
2008	Ilha Comprida, São Paulo, Brazil	28J	75/17.7/24.2	Prado et al., 2011
2008–2010	Região dos Lagos, Rio das Ostras, Arraial do Cabo, Rio de Janeiro, Rio Grande do Sul, Sergipe, Brazil	87	78/31.9/42.1	Brandão et al., 2013
2014–2015	Pontal do Sul, Paraná, Brazil	31	64.5/33.3/51.6	Vanhoni et al., 2018
2016	Santa Catarina, Brazil	19J/1A	35/-/1.8	Ewbank et al., 2020
2021–2023	Mar del Plata, Argentina	20J	30/-/5.3	Present study

Considering that a parasite typical of cold waters may be strongly affected by the rapid increase in sea temperature undergone in the northern Argentine Sea, the large set of data available on anchovies caught during 1993–1995 (Timi et al., 1999; Timi, 2003), the easy identification of metacercariae inhabiting the vitreous humour of the anchovy eyes, requiring a minimum dissection and the availability of published historical data of adult parasites in Magellanic penguins from Argentina and Brazil, constitute a good opportunity for a historical comparative study. Therefore, the aim of this work is twofold. (a) To corroborate the specific identity of metacercariae from anchovies as *C. physalis* and (b) to assess if changes in parasitism did occur in both fish and seabird hosts and the congruence between them, analysing the possible causes for such changes in a scenario of a rapid regional tropicalization.

## Materials and methods

**Figure 1.**
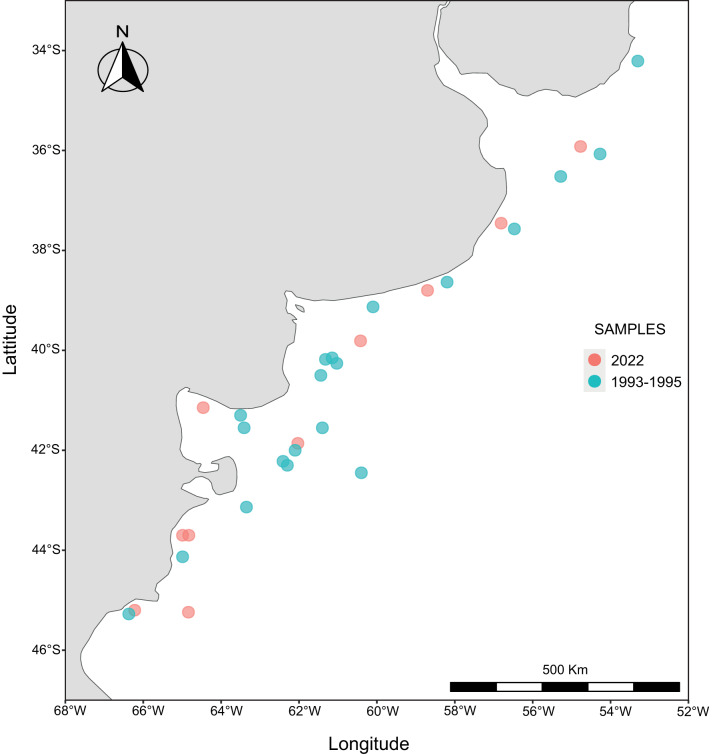
Distribution map of *Engraulis anchoita* samples. Samples from 1993 to 1995 in green, samples from 2022 in orange.

Data on parasitism by metacercariae of *Cardiocephaloides* sp. in the eyes of 1038 Argentine anchovies of different sizes were available from previous studies, most of them unpublished (mainly data about juvenile fish). These fishes were collected during six research cruises of the Instituto Nacional de Investigación y Desarrollo Pesquero (INIDEP) in the period 1993–1995 following a pre-stratified random sampling design and midwater trawl nets were used to collect samples. This sampling scheme covered the southwest Atlantic shelf from 34 to 46º S (Figure 1; Supplementary Material). Anchovies were assigned to four distinct groups or stocks, identified by using the parasite communities of adult fish (total length > 120 mm) as biological tags (Timi, 2003): the autumn north Bonaerense ANB (35–37ºS) and three spring stocks, north Bonaerense SNB (34–40ºS), south Bonaerense SSB (40–43ºS) and Patagonian SP (43–46ºS) (Timi, 2003). Despite both autumn and spring samples from the north Bonaerense zone being caught in the same area, parasitological evidence suggested that autumn samples correspond to a different stock, which probably inhabits northern zones than fish caught during spring in these areas (Timi, 2003). Additionally, and following Angelescu (1982), fish from each stock were grouped into size classes representing the following developmental stages: primary juveniles (36–60 mm total length), secondary juveniles (61–100 mm), pre-adults (101–120 mm) and adults (>120 mm).

Present samples comprise 714 anchovies caught between 35 and 45ºS, from June to December 2022 (Figure 1) by both commercial vessels and during five research cruises of the INIDEP. Fish were assigned to the same stocks as previous ones according to its date and locality of capture. They also were divided into the same size classes. After defrosting, the eyes of each anchovy were examined under a stereomicroscope before and after being removed with the aid of thin tweezers. Metacercariae were preserved in 96° ethanol for molecular identification.

From February to May 2021 (64 penguins) and from February to March 2023 (>40 penguins) were found stranded dead along the beaches of south-eastern Buenos Aires Province. Of these, 20 fresh carcasses were randomly selected for adult *C. physalis* examination. Fresh carcasses included recently dead birds, odourless, thus showing features of rigour mortis and those in slight decomposition, with little odour, but intact internal organs. All sampled birds were identified as young – juvenile – individuals that recently abandoned their breeding grounds, with feathers uniformly coloured, i.e. overall bright coloration with grey-blue on the birds´ back and more faded grey-blue on the chest, with no evident wear (no trace of waxy sheath remaining at the base in spite of fully developed feathers) (see Seco Pon and García, 2022). Whole carcasses were frozen and stored in a sealed plastic bag at−18°C prior to subsequent analysis.

After defrosting, the intestines were dissected out and examined under a stereomicroscope. Adult digeneans were counted and identified.

DNA was extracted from metacercariae using DNeasyTM tissue kit (Qiagen, Hilden, Germany) following the manufacturers’ protocols. For amplification of 28S ribosomal ADN, primers 1500 R (5′-GCT ATC CTG AGG GAA ACT TCG-3′; Snyder and Tkach, 2001) and 300 F (5ʹ-CAA GTA CCG TGA GGG AAA GTT G-3ʹ; Littlewood et al., 2000) were used. PCR reaction was set up in 25-μl reactions using: 5 μl of DNA (≥ 10 ng) as the template, 0.5 μl (10 mM) of each primer, 2 μl (10 mM) of dNTPs, 3 μl (25 mM) of MgCl (Promega), 5 μl (5X) of Green Buffer (Promega), 0.3 μl of Go-Tag Polymerase (5 U/μl) (Promega) and sterilized distilled water up to 25 μL. The following thermocycling profile was used for amplification: denaturation (94°C for 2 min); 35 cycles of amplification (94°C for 30 s, 57°C for 45 s and 72°C for 2 min); and final extension at 72°C for 7 min. Amplified PCR products were verified in a 1.2% agarose gel. The PCR product was purified using QIAquick Gel Extraction Kit or QIAquick PCR purification Kit (Qiagen, Hilden, Germany). Sequencing of both strands was carried out using ABI 3730XLs automated sequencer (Applied Biosystems, Macrogen, South Korea). Sequences were edited and assembled in Proseq v.3.5 (Filatov, 2002) and deposited in the GenBank database. For identification, the generated sequence was compared against the NCBI database using the BLAST algorithm (Sayers et al., 2023).

The prevalence, mean intensity and mean abundance of *Cardiocephaloides* sp. were calculated, according Bush et al. (1997) in each sample of anchovies based on the geographical location and season of collection for both past and present samples, as well as for adult worms in penguins.

To test the occurrence of temporal changes in parasitism, the effects of period, but also of other potential sources of variability, such as total length (LT) and anchovy stock on the abundance of *C. physalis*, generalized linear models with Negative Binomial error distribution, were applied (Crawley, 2007). Obtained models were evaluated with information-theoretic procedures (Burnham and Anderson, 2002). The Akaike’s (AIC) information criterion was calculated for each model (Burnham and Anderson, 2002). Model selections were made considering a ΔAIC < 2. The relative likelihood that a specific model is the best of the suite of all models was determined by the AIC weight (wi). The percentage explained by the model was calculated using the formula: Null deviance-Residual deviance/Null deviance. Deviance percentage of each variable was calculated on the basis of Analysis of Variance with formula: deviance of variable/sum of variance. Statistical analyses were carried out using R software, Version 2.13.1 (R Development Core Team 2011). All tests were two-tailed, and differences were considered significant at *p* < 0·05.

## Results

A total of 309 metacercariae were found in the 1038 anchovies caught between 1993 and 1995. Parasites were found in fish from spring samples only. On the other hand, only four larvae were found in four out of 714 anchovies caught in 2022.

A unique sequence (924 pb) was obtained from the four amplifications performed on the metacercariae found parasitizing anchovies from 2022, probably due to a poor condition of DNA because fish samples were preserved frozen. The molecular characterization of the metacercaria allowed to identify this specimen as belonging to *Cardiocephaloides physalis*, matching with a percentage of identity of 99.89% with sequences of metacercariae found in fishes, *Clinus superciliosus* (MW370426 and MW370427) from South Africa, and penguins, *Spheniscus demersus* (MW370425) and *Spheniscus magellanicus* (MN820665), from South Africa and Chile respectively (Vermaak et al., 2021). The new sequence is available in GenBank under accession number ID: PV110189.

In the older samples, no parasites were found in anchovies caught during autumn whereas a tendency to increase with anchovy size as well as with latitude was evident in terms of both prevalence and mean abundance (Table 2) for spring samples, although no relationship between them. The model that best explained the variation in abundance of *C. physalis* in anchovies included the time period, total length and stock as explanatory variables (wi = 0.9864), explaining the 51% of variation (Table 3). Other models resulted with a ΔAIC > 2 and lower wi (Table 3). The abundance of *C. physalis* in *E. anchoita* decreased significantly in the recent samples (2022) (Table 4), the observed differences of abundance across stocks and its increase with fish size are mainly due to the values of parasitism recorded during the first period. However, when all variables are considered, the most important in determining changes in abundance was the period (Table 5).

**Table 2.** Number of examined hosts (N), prevalence (P) and mean abundance (MA), a *Cardiocephaloides physalis* parasitizing *Engraulis anchoita*. (N/P/MA)

		Size classes			
Stock	Period	30–60 mm	61–100 mm	101–120 mm	>120
ANB	1993–1995	30/0/0	254/0/0	40/0/0	47/0/0
	2022	–	–	19/0/0	50/0/0
SSB	1993–1995	–	93/0.18/0.32	71/0.18/0.25	250/0.44/0.48
	2022	–	33/0/0	22/0/0	134/0.01/0.01
SNB	1993–1995	–	12/0/0	37/0.03/0–08	126/0.07/0.11
	2022	–	27/0/0	14/0/0	39/0/0
SP	1993–1995	–	–	–	78/0.27/1.14
	2022	–	–	8/0/0	368/0.01/0.01

**Table 3.** Summary of model-selection results for models explaining variation in abundance of *Cardiocephaloides physalis* in relation to period of time and total length (LT). models are listed in decreasing order of importance

	LogLik	χ^2^	Pr(>χ^2^)	ΔAIC	df	Weight (wi)
Abundance ∼ Period + LT + Stock	−505.947	–	–	0	7	0.986
Abundance ∼ Period + Stock	−511·929	11·964	0·0005	9·948	6	0·0068
Abundance ∼ LT	−617·981	212·103	1·022e−4^5^	9·948	6	0·0068
Abundance ∼ Period	−585·453	65·056	0·00032	150·960	3	1·634e^−33^
Abundance ∼ Stock	−511·929	147·047	1·1421e^−31^	216·017	3	1·22e−4^7^

**Table 4.** Parameter likelihoods, estimated SE and 95% confidence interval limits (CL) for explanatory variables describing variation in abundance of *Cardiocephaloides physalis* parasitizing *Engraulis anchoita*

Explanatory variable	Parameter likelihood	Estimate std. error	CL	
			Lower	Upper
(Intercept)	−4·813	−2·738^e+01^+/−2·034e^+04^	−6·0412	−3·5842
Period(b)	−4·421	−4·709^e+00^+/−5·561e^−01^	−5·893	−2·950
LT	0·011	1·370e^−02^+/−4·106e^−03^	0·005	0·018
Stock SNB	1·435	2·418e^+01^+/−2·034e^+04^	0·056	2·815
Stock SSB	3·016	2·530e^+01^+/−2·034e^+04^	1·789	4·243
StockSP	3·098	2·612e^+01^+/−2·034e^+04^	1·7824	4·415

**Table 5.** Mixed-model analysis of variance (ANOVA) table to assess different effects on abundance of *Carciocephaloides physalis* parasitizen *Engraulis anchoita*

	Df	% Deviance	Resid. df	Resid. dev	Pr(>χ^2^)
NULL	–	–	1752	713·808	–
Period	1	47·01	1751	542·427	3·694e^−39^
LT	1	24·44	1750	453·344	3·785e^−21^
Stock	3	28·55	1747	349·263	2·059e^−22^

**Figure 2.**
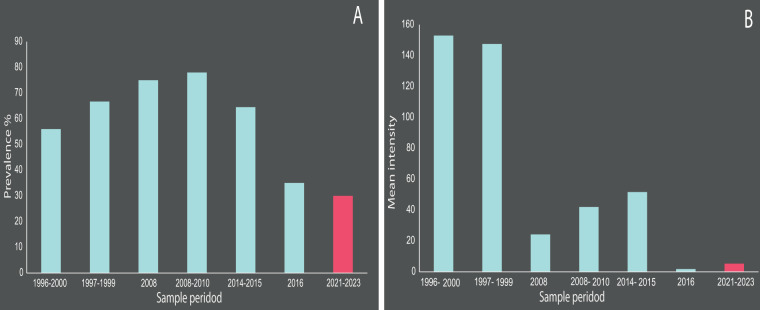
Prevalence (A) and mean intensity (B) of *Cardiocephaloides physalis* from penguins *Spheniscus magellanicus.* Samples from 1996 to 2016 in light blue, samples from 2021 to 2023 in red.

The values of parasitism recorded in the present samples (2021–2023) of penguins also showed low values of prevalence (Figure 2a) and mean intensity (Figure 2b) relative to previous data from literature, especially with those from 1999 to 2000, but similar to those recorded in a sample from 2016 (Figure 2, Table 1). Penguins examined between 2008 and 2015, on the coast of Brazil, showed intermediate values.

## Discussion

The present work confirms the identity of metacercariae found in *E. anchoita* as *C. physalis*, as has been suggested in previous works (Timi et al., 1999; Timi, 2003), providing the first molecular confirmation of this species for the Southwest Atlantic Ocean and highlighting its broad distribution in the Southern Hemisphere.

Three decades ago, *C. physalis* was relatively common in the eyes of adult anchovies (Timi et al., 1999; Timi, 2003), and according to the present analyses of old data, also for juvenile fish, except for primary juvenile ones. This would explain the relatively high values of prevalence and abundance of adults *C. physalis* in penguins during that period, given the predominance of anchovies in the diet of penguins living north of 45ºS (Yorio et al., 2017; Ciancio et al., 2018; Blanco et al., 2022). In those times, the trematode followed the cumulative pattern commonly displayed by long-lived larval parasites in intermediate and paratenic hosts (Braicovich et al., 2016), increasing in number with fish size. In this case, as strigeid cercariae actively penetrates the skin of secondary hosts (van Beest et al., 2022), the transmission success under natural conditions relies mostly on host encounter rates and not on the feeding activity or amount of fish. Therefore, a differential use of habitat between juvenile and adult anchovies, and consequently of the encounter rates with cercariae, could explain the cumulative pattern plus the fact that older fish have been exposed for longer. The relevance of host size was also clear considering the percentage of deviance in the selected model, a pattern surely determined by the value of parasitism in the first period. Similarly, the effect of host stock was due to metacercariae found in 1993–1995. Indeed, the same geographical pattern was also evident three decades ago, with an increasing number of parasitized hosts and of parasites per fish with latitude, indicating a preference for colder waters. Likewise, metacercariae found in the eyes of *S. sagax*, in South Africa, showed significantly higher values of parasitism in sardines associated with the cold Benguela Current regarding the warmer Agulhas Current (Weston et al., 2015).

The conspecificity of larvae and adults *C. physalis* could also explain the concomitant decrease of both stages in intermediate fish and definitive bird hosts, with only larger fish from southern latitudes harbouring the few worms currently recorded. Unfortunately, no data of parasitism in anchovies at intermediate dates were available. Therefore, we are reporting just ‘snapshots’, which do not provide complete evidence of the entire process (Mushet et al., 2019). However, the gradual decline of the population of adult parasites in Magellanic penguins over the last three decades, related to the concomitant decrease of their larvae is an expected result.

Assigning a causative relationship to the observed decrease in parasitism would be speculative, because many interacting variables, biological and environmental, could be acting simultaneously. The widespread effects of environmental change on the ocean biota during recent decades (Carlson et al., 2017; Tracy et al., 2019) have a series of possible causes, some of them interdependent from each other, including, in the case of host-parasite systems, the ecophysiology of their interactions and shifts in behaviour, movement and phenology of both hosts and parasites (Altizer et al., 2013). However, some possible causes and their respective probabilities of occurrence will be explored here, including changes in host densities, host-parasite encounter rates, and a direct effect of environmental change on parasites.

Any parasite species requires densities of both definitive and intermediate hosts large enough to sustain viable populations, as well as suitable to optimal environmental conditions for their development, reproduction and transmission. Changes in these variables could explain the observed pattern. A wide variability in abundance is a typical feature of small pelagic fishes compared with other fish taxa (Hilborn et al., 2022), particularly for engraulids (Pennino et al., 2020). However, no evidence of drastic changes in the density of Argentine anchovies has been recorded during the last decades (Orlando et al., 2024a, 2024b), although their success in recruitment undergoes interannual variations related to fluctuations in chlorophyll concentration influenced by changes in water temperature and vertical stratification (Marrari et al., 2013). Indeed, anchovies have been catalogued as those with the lowest sensitivity to climate change among fishery resources, although with the highest potential to adjust its distribution (Gianelli et al., 2023) in response to changes in the regional warming hotspots (Alvarez Perez and Sant’Ana, 2022). Similarly, the overall population size of *S. magellanicus* in both southern and northern regions of the distribution of breeding colonies in the Atlantic coasts has remained relatively stable over the last 25 years (Millones et al., 2021; García-Borboroglu et al., 2022). Indeed, in northern Patagonia (Chubut and Rio Negro provinces, Argentina), which is the stronghold of the known global population, trends are mixed. Whereas the largest colonies are declining in the central and southern part of northern Patagonia (Boersma et al., 2015; Pozzi et al., 2015; Braicovich et al., 2016; García-Borboroglu et al., 2022), the breeding population has expanded north since the 1960s, with new colonies established and growing rapidly (Schiavini et al., 2005; Boersma et al., 2015; Pozzi et al., 2015). Climate change, however, through increases in the frequency and intensity of storms has resulted in reproductive failure of Magellanic penguins, lowering their reproductive success and undermining their resilience (Boersma and Rebstock, 2014). Unfortunately, the first intermediate molluscan host of *C. physalis* is unknown and, therefore, possible changes in its populations cannot be disregarded.

As parasites depend on other species for transmission, phenological mismatches between hosts and parasites can alter the severity of diseases due to their differential responses to rising temperatures, leading to reductions in disease (Paull and Johnson, 2011). Consequently, it is possible that the geographic distributions of many parasites may actually experience net declines with climate change. During the breeding season, the diet and foraging behaviour of *S. magellanicus* are variable along its distribution range in Atlantic waters, in terms of both prey composition and size (Wilson et al., 2011) and any environmental change could cause a shift in the distribution of anchovies, which would affect the foraging behaviour of penguins (Blanco et al., 2022) and therefore the probabilities of acquiring parasites. Unfortunately, no information is available to assess at the proper spatial scale possible changes in the availability of Argentine anchovy in the study region and its potential effect on the diet and foraging behaviour of Magellanic penguins (García-Borboroglu et al., 2022). On the other hand, a progressive northward shift in the septentrional limit of the Atlantic distribution of breeding colonies of *S. magellanicus* has been taking place over several decades (García-Borboroglu et al., 2022). Owing that all examined birds were juvenile ones, they could have come from these northern colonies, where they could be fed with ‘northern’ and consequently less parasitized anchovies caught by their parents during the chick-rearing period. Nevertheless, both adult and juvenile penguins can potentially track patches of Argentine anchovy for long periods and follow its northward migration from their breeding grounds in Argentina to their wintering grounds in southern Brazil (Marques et al., 2018) where the most important factor explaining the density of penguins at sea is the anchovy density (Costa et al., 2020). For such a reason, it is little probable that phenological mismatches or a decrease in host-parasite encounter rates are the main cause of the observed decline of parasite population.

Finally, ectothermic hosts and parasites with environmental transmission stages that can survive outside the host provide the best examples of infectious disease responses to climate change, because their rates of development and transmission should be more sensitive to temperature than other host-pathogen interactions (Altizer et al., 2013). Trematodes such as *C. physalis*, with free-living eggs, two free-swimming larval stages, such as miracidia and cercariae, and two ectothermic hosts, such as mollusks and fishes, are highly susceptible to changes in sea temperature, which can affect the release, embryonic development and hatching of eggs, the longevity and infectivity of free-living stages and the development, maturation, longevity and mortality of adult parasites (Marcogliese, 2001; Berkhout et al., 2014; Selbach and Poulin, 2020). Therefore, a direct effect of an increase in sea temperature on parasites arose as the main causal candidate for the decline in parasite populations occurred along the last decades. Furthermore, whereas the effects of global change on parasitism have been mostly studied experimentally concerning to temperature (Marcogliese, 2016), parasites can respond directly to changes in other climate-driven abiotic parameters, such as salinity and UV radiation, which can have their own and/or combined effects on parasite survival (Studer and Poulin, 2013).

At present, the negative impacts of climate change on parasite diversity are largely undocumented (Carlson et al., 2017). With climate change prospects predicting at least 4°C of global warming by the end of this century (Parry et al., 2009; Stafford Smith et al., 2011), the multiplicity of developmental stages, including free-living and parasitic phases, make many parasites especially susceptible to these environmental threats (Marcogliese, 2001; Sures et al., 2023). This is particularly true for those parasites with high host specificity, complex life cycles or those that infect ectothermic hosts during one or more life cycle phases, which are at greatest risk (Harvell et al., 2002; Cizauskas et al., 2017). However, temperature effects on the physiological homeostasis of endotherm hosts, predominantly on endocrine and immune systems, may also have deep implications for parasite epidemiology (Morley and Lewis, 2014). In this sense, beyond the biological and ecological consequences of global change on them, parasitic organisms offer excellent systems for measuring and monitoring such effects. The almost local extinction of *C. physalis* in a marine hotspot of global warming seems to be one of the first examples and a tool for predicting and modelling future environmental changes.

## Supporting information

Marcotegui et al. supplementary material Marcotegui et al. supplementary material

## Supplementary material

The supplementary material for this article can be found at https://doi.org/10.1017/S0031182025000216.
