# Supplementary material for: Local extinction of a parasite of Magellanic penguins? The effect of a warming hotspot on a ‘cold’ trematode
Source: Parasitology. 2025 Feb 27;152(3):275–83. doi: 10.1017/S0031182025000216 (PMC12186095; doi:10.1017/S0031182025000216)
Supplement: Marcotegui et al. supplementary material [file S0031182025000216sup001.docx]

Supplementary

| **Time** | **Origin** | **Position** | **Stock** | **Mean Length** | **N** |
| --- | --- | --- | --- | --- | --- |
| 1993 | Research Cruise | 41.30S; 63.50W | SSB | 103 | 25 |
| 1993 | Research Cruise | 41.55S; 63.41W | SSB | 99.76 | 20 |
| 1993 | Research Cruise | 42.22S; 62.41W | SSB | 98.87 | 16 |
| 1993 | Research Cruise | 43.22S; 63.26W | SP | 148.68 | 25 |
| 1993 | Research Cruise | 44.36S; 65.17W | SP | 160.10 | 29 |
| 1993 | Research Cruise | 45.22S; 66.43W | SP | 161.75 | 24 |
| 1994 | Research Cruise | 37.57S; 56.47W | SNB | 126.83 | 77 |
| 1994 | Research Cruise | 36.07S; 54.27W | ANB | 111.77 | 112 |
| 1994 | Research Cruise | 36.52S; 55.28W | ANB | 77.95 | 112 |
| 1994 | Research Cruise | 34.21S; 53.30W | ANB | 82.61 | 148 |
| 1994 | Research Cruise | 40.15S; 61.15W | SSB | 163.30 | 102 |
| 1995 | Research Cruise | 38.50S; 58.43W | SNB | 140.42 | 40 |
| 1995 | Research Cruise | 39.13S; 60.10W | SNB | 134.84 | 58 |
| 1995 | Research Cruise | 40.50S; 61.44W | SSB | 67.09 | 44 |
| 1995 | Research Cruise | 40.26S; 61.03W | SSB | 134.79 | 49 |
| 1995 | Research Cruise | 40.18S; 61.32W | SSB | 138.50 | 6 |
| 1995 | Research Cruise | 42.45S; 60.40W | SSB | 141.69 | 42 |
| 1995 | Research Cruise | 41.55S; 61.40W | SSB | 137.52 | 43 |
| 1995 | Research Cruise | 42.00S; 62.10W | SSB | 113.84 | 47 |
| 1995 | Research Cruise | 42.30S; 62.30W | SSB | 138.37 | 19 |
| 2022 | Fishing Ship | 43.70S; 64.99W | SP | 160.37 | 40 |
| 2022 | Fishing Ship | 43.70S; 64.83W | SP | 143.17 | 40 |
| 2022 | Fishing Ship | 38.80S; 58.7W | SNB | 145.17 | 40 |
| 2022 | Research Cruise | 41.86S; 62.03W | SSB | 126.35 | 40 |
| 2022 | Research Cruise | 39.81S; 60.42W | SSB | 139.5 | 40 |
| 2022 | Research Cruise | 37.43S; 56.96W | SNB | 96.55 | 40 |
| 2022 | Research Cruise | 41.01S;64.31W | SSB | 123.06 | 109 |
| 2022 | Research Cruise | 35.92S; 54.77W | ANB | 124.49 | 69 |
| 2022 | Research Cruise | 45.24S; 64.84W | SP | 133.85 | 281 |
| 2022 | Research Cruise | 45.20S; 66.22W | SP | 142.2 | 15 |
